# Supplementary material for: Content-rich biological network constructed by mining PubMed abstracts
Source: BMC Bioinformatics. 2004 Oct 8;5:147. doi: 10.1186/1471-2105-5-147 (PMC528731; doi:10.1186/1471-2105-5-147)
Supplement: Additional File 5 — The original Chilibot query results of the term "long-term potentiation (LTP)" and 22 other terms, limiting the latest references analyzed to the years 1990, 1995, 2000, and 2004. [file 1471-2105-5-147-S5.bz2 › chilibotAdditionalFile5/ltp1995/html/ACTIN_SYNAPTOPHYSIN.html]

 


 **ACTIN** and **SYNAPTOPHYSIN** 
  
Found 36 abstracts in PubMed,  **30 abstracts were retrieved and analyzed**.  


---

 Search Google  |
 PDF files only 
|  EDU domain only 

---

**Interactive relationship** (e.g. stimulation, inhibition, etc)

**Parallel relationship** (e.g. studied together, co-existance, homology, etc.)

- All tumors were negative for leukocyte common antigen, muscle specific  **actin** , and  **synaptophysin** .  Ref: 1720920 Am J Clin Pathol, 1991
- Antibodies against cytokeratin, desmin, vimentin, neurofilaments, neuron specific enolase, Leu7,  **synaptophysin** , chromogranin, the muscle specific  **actin** , the Von Willebrand factor and the common leucocyte antigen were used in a biotin streptavidin procedure.  Ref: 8516214 Pathol Res Pract, 1993
- Both types of tumor giant cells stained for vimentin, alpha 1 antitrypsin, alpha 1 antichymotrypsin,  **synaptophysin** , muscle  **actin** , and neuron specific enolase, but not for epithelial markers.  Ref: 2227926 Hum Pathol, 1990
- All 12 ITAC were negative for vimentin,  **synaptophysin** , and  **actin** .  Ref: 7567943 Mod Pathol, 1995
- The expression of keratins, vimentin, neurofilament proteins, smooth muscle  **actin** , neuron specific enolase, and  **synaptophysin**  in tumors of the specific glands in the canine anal region.  Ref: 8212457 Vet Pathol, 1993
- Paraffin immunostaining results were number positive total vimentin 8 9, NSE 10 10, S100 protein 6 10, neurofilament protein 0 9,  **synaptophysin**  3 9, desmin 2 9, focal, smooth muscle  **actin**  0 9.  Ref: 7892047 Pathology, 1994
- Antibodies against acetylcholinesterase,  **synaptophysin** , alpha smooth muscle  **actin** , calcitonin gene related peptide CGRP, neuropeptide Y NPY, nitric oxide synthase NOS, substance P SP, tyrosine hydroxylase TH, and vasoactive intestinal peptide VIP were used.  Ref: 7601644 Invest Ophthalmol Vis Sci, 1995
- This pattern resulted from binding of the antibodies to synaptic complexes of both rods and cones, shown by double labelling with antibodies to either  **synaptophysin**  or  **actin** .  Ref: 8163390 Histochemistry, 1993
- None contained desmin,  **actin** , cytokeratin, epithelial membrane antigen, or  **synaptophysin** .  Ref: 2202275 Arch Pathol Lab Med, 1990
- The present study is the first consecutive analysis of the adult normal human organ of Corti and vestibular hair cells with regard to the expression of F  **actin** ,  **actin**  associated proteins alpha actinin, alpha and beta spectrins, vinculin and tropomyosin, beta tubulin and the calcium binding protein  **synaptophysin** .  Ref: 7731659 ORL J Otorhinolaryngol Relat SpecORL J Otorhinolaryngol Relat Spec, 1990
- In an attempt to address this issue, we performed an immunohistochemical analysis of nine cases, using antibodies to vimentin, S100 protein, Leu 7 antigen, neuron specific enolase,  **synaptophysin** , desmin, muscle specific  **actin** , cytokeratin, and epithelial membrane antigen, and the avidin biotin peroxidase complex ABC method.  Ref: 2202275 Arch Pathol Lab Med, 1990
- No positive reactions were obtained with antibodies against heat shock protein 27 HSP 27, HSP 72,  **actin** , vimentin, desmin, cytokeratin, myelin basic protein, beta tubulin, MAP 2, tau protein, paired helical filament, phosphorylated neurofilament protein NFP, nonphosphorylated NFP,  **synaptophysin** , cathepsin D, alpha 1 antichymotrypsin, alpha 1 antitrypsin and basic fibroblast growth factor.  Ref: 1332366 Acta Neuropathol (Berl), 1992
- **Synaptophysin** , chromogranin, muscle specific  **actin** , and glial fibrillary acidic protein were absent.  Ref: 1384369 Am J Surg Pathol, 1992
- GISTs were immunopositive in the following categories vimentin 45 of 46, desmin nine of 45, muscle specific  **actin**  MSA 36 of 46, alpha smooth muscle  **actin**  SMA 34 of 46, chicken gizzard  **actin**  7 zero of 38, cytokeratin two of 46, S100 protein six of 46, glial fibrillary acidic protein GFAP zero of 46,  **synaptophysin**  zero of 46, and chromogranin one of 46.  Ref: 1415903 Am J Surg Pathol, 1992
- The monoclonal antibody KP 1 that recognizes the lysosome associated glycoprotein CD68 was used together with antibodies to other antigens  **actin** , glial fibrillary acidic protein, keratin, neurofilaments, chromogranin,  **synaptophysin** , S 100 protein, HMB 45, lysozyme, and HLA DR in a labeled streptavidin biotin immunoperoxidase method to phenotypically characterize 27 granular cell tumors, five schwannomas, five neurofibromas, two ganglioneuromas, three ganglioneuroblastomas, five carcinoid tumors, five malignant melanomas, and five examples of histiocytosis X.  Ref: 7959661 Hum Pathol, 1994
